# Oleum Animale

**Published:** 1887-08

**Authors:** Julius Schmitt

**Affiliations:** Rochester, N. Y.


### OLEUM ANIMALE.

### OLEUM CORNU CERVI (DIPPEL'S OIL).

JULIUS SCHMITT, M. D., ROCHESTER, N. Y.†

*Mind.*—Sad thoughts force themselves on him and make him very ill-humored.

Morose, fretful.

Dreamy state, thoughts vanish.

---

† Read before the Hahnemannian Society of Rochester.

Sudden loss of consciousness; this lasted only a moment at one P. M.

*Sensorium.*—Vertigo and dizziness in stooping *in the open air.*

*Head.*—*Sticking,* tearing, burning, PRESSING headaches, RELIEVED BY RUBBING.

*Worse* from slight mental exertion (forehead), AFTER and during dinner, in open air; and at seven P. M., during menstruation.

Fine itch like an electric spark in the left frontal eminence at seven P. M.

Fine drawing with a cold sensation in the left temporal region.

Left side of the head seems numb and paralyzed.

On going into the house it seemed as if the blood suddenly rushed into the occiput.

Pressure in left side of the occiput obliges him to hold the head forward, lasting from an hour after dinner till six P. M.

*Outer head.*—Tension of the occipital muscles.

Tearing in three different places on the scalp at the same time; afterward a tension with a feeling of soreness, as if the skin had been cut and reunited.

Itching here and there on the scalp; relieved by scratching.

*Eyes.*—Twitching in left eyebrow, lasting half an hour, followed by a sensation as if the skin were hanging down and preventing vision, at six P. M.

Twitching of left upper lid.

Eye symptoms are relieved by RUBBING.

Worse while eating and after dinner; in open air; in the afternoon and evening (writing).

Fine biting, with stitches like electric sparks in both eyes.

Agglutination of eyelids.

(Lachrymation during eating [dinner].—Berridge.)

Shortsightedness.

A fog before the eyes, and a sensation as if many small, glistening bodies were moving back and forth before the vision at two P. M., while writing.

*Ears.*—Burning in left ear, extending from within outward, lasting a quarter of an hour, at half-past five P. M.

Tearing in front of the ears.

Heat coming out of right ear.

Itching in ears; relieved by rubbing.

Tones reach the ear as though a great noise.

Loud noises increase the roaring in the ears.

Cracking, singing, and ringing in left ear.

Relief of symptoms from rubbing appear at two, half-past two, half-past five, a quarter of six, and five P. M., and eight P. M.

*Nose.*—Nasal mucus rapidly becomes thick and profuse, and causes tension and pain in the nose.

Stoppage of nose.

Sneezing, with sore or violent bursting pain in the chest.

Tickling in nose, mostly left side; relieved by rubbing.

Great irritation to sneeze in right nostril.

Sensation of prickling as from acrid vapor in the upper part of the nose.

It seems as though he had an offensive breath.

Symptom at two or half-past two P. M., an hour after dinner (?) and at seven P. M.

*Face.*—Color of the face pale, almost earthy, all the afternoon.

Tensive spasmodic drawing in the face.

Paralytic sensation in either half of the face.

Redness of cheeks, without internal or external sensation of heat, even with a cold skin.

Crawling.

Burning, especially about the chin, every morning, followed by desquamation.

A sensation in both malar bones as if one pulled them forcibly upward.

Lips cracked.

Twitching in both lips, so that he woke during morning nap.

Swelling beneath the right lower jaw, so great that the skin was tense; a drawing with pain extending to the ear.

Violent cracking in left articulation of jaw; always on opening the mouth.

Cramp in the lower jaw at half-past six A. M.

Symptoms relieved from rubbing.

*Mouth and Teeth.*—Drawing, tearing stitches in teeth; removed by pressure after dinner.

Jerking and tearing in the root of a right lower hollow tooth, frequently throbbing like an ulcer from afternoon till evening, though frequently intermitting with a sensation of icy coldness coming out of the tips of the teeth.

*Tongue.*—Biting on the tongue posteriorly, as from tobacco.

*Sour*, fatty taste.

Profuse accumulation of saliva, as white as snow.

Pricking on the palate posteriorly, lasting a long time, at two P. M.

*Throat.*—Rawness, burning, dryness of throat.

Burning on right side of throat.

Rawness in a long streak in the throat that provokes a short cough (cough causes crawling on left side).

Almost constant dryness of the throat, especially noticed on empty swallowing; this is relieved for a long time by eating.

Dryness in the morning with a sensation as if cold air penetrated, which she was constantly obliged to swallow; swallowing was very difficult, though food and drink passed very easily.

Sensation of an acrid vapor in the throat.

Burning in pharynx as from alcoholic drinks or as from pepper, extending gradually to the stomach.

When lying on the back with the head inclined forward upon the chest there is a sensation as if something compressed the larynx, almost stopping the breath, and only disappearing on changing the position.

Sensation in the throat on empty swallowing as if a foreign substance were hanging down; it seems as if it could be raised by hawking, with ineffectual attempts for a long time till it provoked vomiting, when two pieces of a thick brown glutinous substance, as large as hazelnuts, were forcibly ejected, after which the dryness on swallowing disappeared for a short time at eight A. M.

*Appetite and thirst.*—Wants only bread.

Desire *for soft eggs*, potatoes, and soup.

*Aversion to meat.*—Thirst in the evening.

*Hiccough, nausea, and vomiting.*—Violent hiccough, nine P. M.

Eructations tasting of the drug or urine.

Empty eructations during dinner.

Nausea during or after dinner; better after coffee.

Nausea and pressure upon the chest; sensation as if one had swallowed too big a morsel, and rising of gases toward the throat, almost as in heartburn.

Nausea, with constrictive pains in intestines extending to the stomach, with dry cough and stitches beneath the sternum.

Sudden inclinations to vomit, the stomach seems to turn over; after eructations twice it disappears.

*Stomach.*—Burning, distention, coldness, emptiness, twisting, pressure in stomach, distention in stomach and abdomen, with frequent eructations and emissions of flatus.

Sensitiveness to pressure.

Rumbling followed by eructations.

Pressure in stomach and œsophagus relieved by eructations, followed by offensive exhalations, at times with, at times without, eructations—relieved by pressure—after drinking fresh water; *worse in open air.*

*Painful stitches* in epigastrium three or four times with rising of warmth and disappearance of the coldness.

*Some dull stitches* in succession in *left side* of epigastrium, and immediately afterward under the *left* mamma.

Bruised sensation in left side of stomach, with pain on pressure, *disappearing on rubbing,* a quarter of an hour *after eating.*

Sensation as if the stomach were filled full of water up to the throat.

Heat as if fire were in the stomach, extending into the pectoral region.

Feeling of coldness in the stomach, as if ice were lying in it.

Acute fasting sensation in stomach.

Stomach and abdomen seem eviscerated in the morning.

*Hypochondria.*—Pain as if ulcerated in *right* hypochon.; on ceasing there, is extended *to the left* side.

*Sticking,* pressive pain in region of liver and spleen.

*Dull stitches* in hepatic region, *on walking in the open air.*

*Abdomen.*—Flatulency with cutting and griping, relieved by passage of flatus.

Offensive flatus. Rumbling and movements in the abdomen. Symptoms worse *after supper*—after warm food and drink.

Movements in the abdomen extending below left breast, almost like a bubbling, as if diarrhœa would occur; half an hour after dinner.

Awakened by cutting pains in the whole abdomen at four A. M., followed by a liquid stool without relief; she had two stools within ten minutes, after which the pains were relieved.

A sudden *fine* cutting pain around abdomen about the umbilicus, as if a sabre were drawn from the left to the right side.

Painful stitch (piercing) deep in the left side of abdomen opposite to the navel, extending like lightning to the right side and making her start at two P. M.

Hot risings from the abdomen into the chest.

Bowels are painful, as if beaten, after stool, at seven A. M.

Painfulness of intestines, as after long constipation, on every motion of the trunk, with great distention of abdomen.

Burrowing and cutting in hypogastrium (by eating and drinking, walking, standing), by sitting.

Acute drawing from the inguinal region into the testicle of the same side, in the left and right side alternately (*Raue's Record*, 1870, page 241).

*Rectum and Anus.*—Burning and stitches in rectum.

Stitches in Anus.—pressing in anus after stool.

Itching in anus from scratching.

*Stool.*—Diarrhœa in the evening, preceded and followed by cutting in abdomen; after the stool burning like fire in the anus.

Constipation, stool evacuated with great efforts, sometimes ineffectual desire to stool.

*Urinary Organs.*—Pressure upon the bladder; burning of urethra during micturition; itching in the urethra; frequent micturition in afternoon, though but little at a time; urine pale, urine greenish, urine becomes turbid over night, like muddy water, and deposits a clay sediment, urine deposits a thin cloud.

*Sexual Organs; male.*

Acute drawing in upper portion of penis in the evening.

Sticking cutting pain in the penis.

Burning stitches in root of penis at three P. M.

Itching on the penis, close to the scrotum, at two P. M.

Testicles are swollen and painful to touch alternately.

Swelling and painfulness of right testicle.

Drawing pain in left testicle.

Testicles retracted, painful.

As soon as he falls asleep again, in the morning, he has erections and emissions in the morning.

*Female.*

Thin, white leucorrhœa.

Before menses, cutting in abdomen and small of the back.

Menses too early, with diarrhœa and weakness of hands and feet, followed by headache like a sticking in left side of head and vertex from seven P. M. until morning, ceasing after rising.

*Respiratory Organs.*—Spasmodic constriction in the trachea, after disturbing sleep, at night. (See *Raue's Record*, 1870, page 157.)

Dry, hacking cough, expectoration after breakfast and dinner, at two P. M.

A frequent sensation as if caused by acrid smoke.

*Chest.*—Violent pressure in the upper part of the chest, extending to between the shoulder-blades, at two P. M.

Oppression of the chest, on ascending a height, on account of great distention of the abdomen; greatly relieved by the emission of flatus, three P. M.

Anxiety in the chest, with frequent shivering, all the forenoon.

Bubbling as from a liquid or as from a spasm, extending from the middle of the chest to the stomach.

Sticking pains beneath the left ribs, aggravated by stretching up the body.

A violent stitch in the upper part of the right side, near the sternum, as from a glowing hot needle; the breast continued to burn for a long time, at two P. M.

A stitch in the right clavicle.

A fine stitch in the middle of the left clavicle, followed by a swinging sensation.

Some dull stitches beneath left mamma that disappear on rubbing, but return; at the same time fine tearing in the right ring and middle fingers.

A pain, almost like a sticking, beneath and behind left mamma; rubbing, followed by warmth over the whole body.

*Heart and Pulse.*—Pressure and bruised feeling.

Pulse small or full and slow, fifty-five to sixty-five beats.

*Neck and Back.*—Hard pressure with stiffness of the neck; must bend head forward; in afternoon.

Cracking in the joints of the nape on raising the head (Natr. c., Nitr. ac., Stan., Sulph., Thuj., Aloes at eight A. M., Nux vomica).

Tearing in right side of neck, extending to right malar bone, and in two right upper molars.

Sensation as if a warm breath extended up the nape, with an agreeable feeling.

Drawing and stiffness in right cervical muscles, not aggravated by motion but by touch.

Sudden sticking in the region of the right lower false ribs by the spine; the place is also sensitive to pressure (Sep.).

A sharp stitch between the shoulders, more to the left and repeated in bending the arm backward to point out the spot, *worse after dinner.*

Burning on the upper margin of the left scapula from rubbing; stitches in left scapula; pain in small of back; as if sprained, when stooping; while sitting.

*Extremities.*—Paralyzed sensation in left arm and left leg.

Tearing, drawing pain in the knees, left shoulder, upper arm, and left side of chest.

*Upper Extremities.*—Powerlessness of both arms as far as the fingers, with tearing, drawing pains.

Rheumatic pains alternately in the scapulæ, shoulders, cervical and pectoral muscles, but worse in the right cervical muscles and right scapula; several days.

Rheumatic pains in the shoulders, muscles of the shoulders, and nape of the neck, in the morning in bed, disappearing after motion.

Tearing in the anterior surface of the right upper arm, with yawning.

Tearing in the outer or inner condyle.

Violent, painful tearing extending from the bend of the elbow to the wrist, along the outer side toward the little finger, in which the pain is most severe; *on rubbing the pain is relieved* everywhere, except in the joints.

Slight tearing extending from the inner surface of the middle of the forearm to the middle of the wrist, *disappearing on rubbing.*

Burning on the outer surface of the right forearm, disappearing on rubbing, at one P. M.

A pinching transversely across the back of the left hand, on allowing the hand to hang down in the evening while walking.

Tearing in all the fingers toward the back of the hand; *on washing the hand in cold water* it disappears after drying them; numbness in different fingers.

Ulcerative pain in the right little finger under both sides of nail, both by itself and especially when pressed upon, in the morning waking, lasting all day, with pressive headache that disappears on rising and walking about.

Tearing, fine tearing, burning, bruised pains in upper extremities, worse after dinner and in open air; better from rubbing, jerking. Jerking, tearing, and fine and sharp stitches in the fingers; pains going downward.

*Inferior Extremities.*—Drawing pain in the right leg, by talking or motion; tearing in the hips.

Acute drawing in the left trochanter.

Dull stitches in the left natis at half-past three P. M.

Soreness as if denuded in the bend of the knee.

Tension in the hollow of left knee as if the tendons were too short.

Tearing in the right knee, with ulcerative pain; *on rubbing* the tearing at first disappears and afterward the pain.

Spasmodic drawing from the hollow of the knee to the back of the foot.

Pain, like a growing-pain, below both knees, frequently repeated during the day, especially in the morning.

Tearing in the whole left leg, from the knee, where the pain is most severe, downward, so that the whole left leg trembles.

Sensation as if the blood pressed forcibly into the lower portion of the left foot, especially into the great toe, at half-past six P. M.

Pains, like an ulcer, in the left great toe, especially by the nail, on pressure, with itching of the nail.

Tearing, drawing, heaviness, stiffness, and weariness in lower extremities.

Trembling of lower extremities, or only of his feet, while standing, better when sitting.

Left side seems to be more affected.

Symptoms appear after dinner and P. M.

Pains going downward. Relief from rubbing not so pronounced.

*Generalities.*—Exhaustion, weakness, and indolence, and discomfort especially after dinner.

Perspiration, saliva, urine, and fæces smell of the drug.

*Skin.*—A pimple here and there in the face and in the bend of right elbow.

Two blisters beneath the skin of the occiput, with a sore pain, aggravated by touch.

Itching almost in every part of the skin of the whole body, relieved by scratching.

Formication, extending from the knee over the whole leg, even to the toes, as if the leg would fall asleep, relieved by rubbing.

Violent formication on the left leg, on sitting down after standing, with a sensation of great weakness.

Itching noticed in the afternoon and evening like all the other symptoms.

*Sleep.*—Yawning and stretching, with sleepiness, disappearing in the open air.

Great sleepiness after dinner, disappearing in the open air.

Could fall asleep at his work, and very sleepy at four P. M.

Restless sleep; falls asleep late and wakes early.

Slightest noise disturbs sleep.

Extremely pleasant dreams of a beautiful region.

Dream of dead persons; dream of murders.

*Fever, Chilliness.*—Shaking chill with goose flesh from half-past seven to nine P. M. before lying down, not in bed, preceded by thirst.

Chilliness, with cold hands, head, and redness of face.

Chilliness in the whole left lower extremity.

Shivering on entering a warm room, even near the hot stove.

Shivering over the scalp, extending from vertex to the chest.

Coldness of the hands, followed by warmth and crawling in the palms.

Coldness of whole left lower extremity.

Coldness of feet, especially of the right one.

Icy-cold sensation in different toes.

*Heat.*—Sensation of heat over the whole body, without external heat, at six P. M.

Sudden heat in the head, with perspiration on the top of head, on the forehead and hands, at half-past six P. M.

Heat in head, with a sensation as if warm vapor rose into it, without external heat.

Prickling heat in face.

Transient sensation of heat frequently in various parts of the body, the ear, arm, leg, with chilliness of the body.

Warmth of the whole body, that commences in the abdomen, with anxiety and perspiration in the region of the stomach and chest, after eating soup, lasting ten minutes.

Flashes of heat, with perspiration on the head, chin, hands, soon followed by a sensation as if cold air were blowing on the head, at half-past one P. M.

Perspiration in the morning in bed, with a good condition of body and mind ; perspires easily while eating.

Sweat, without heat on the hands, from time to time, at four P. M.

*Character of Pains Predominant.*—Fine stitches, fine drawings we find under : Head, eyes, stomach, liver, abdomen, rectum and anus, chest (inner and outer), upper extremities.

*Fine stitches like electric sparks.*—Left frontal eminence and in eyes.

*Left side more affected under:* Head, eyes, ears, stomach, extremities, chilliness (whole left leg).

*Pains going from left to right:* Abdomen.

*Pains going from right to left:* Hypochondr., head.

*Pains going downward:* Upper and lower extremities.

*Cold sensations:* Left temporal region, in tips of teeth, in different toes, in throat, in stomach.

*Prickling as from acrid vapor:* Nose, throat, respiratory organs.

*Numb, paralyzed:* Left side of forehead, left arm, left leg.

*Aggravations.*—Almost all the symptoms appear in the afternoon, at two P. M. and up to nine P. M.

*After dinner:* Headache, eyes, ears, teeth, nausea, stomach, abdomen, cough, back, upper and lower extremities, exhaustion, sleepiness.
*During dinner:* Head, eyes, eructations, nausea, perspiration.
*Open air:* Vertigo, head, eyes, stomach, liver, upper extremities.
*Breakfast.*—Cough.
*Entering house.*—Rush of blood to occiput.
*Stooping.*—Vertigo. Back.
*After supper.*—Abdomen.
*Talking.*—Pain in right leg.
*Washing hands in cold water.*—Tearing of fingers.
*Writing.*—Eyes.
*Yawning.*—Right upper arm.
*Amelioration.*—*Rubbing.*—Headache, scalp, eyes, ear, face, stomach, anus, chest, back, upper extremities; less lower extremities; skin. *Coffee.*—Nausea. *Open air.*—Yawning, stretching, and sleepiness.

